# The evolutionary diversification of LSF and Grainyhead transcription factors preceded the radiation of basal animal lineages

**DOI:** 10.1186/1471-2148-10-101

**Published:** 2010-04-18

**Authors:** Nikki Traylor-Knowles, Ulla Hansen, Timothy Q Dubuc, Mark Q Martindale, Les Kaufman, John R Finnerty

**Affiliations:** 1 Department of Biology, Boston University, 5 Cummington Street, Boston, Massachusetts 02215, USA; 2 Kewalo Marine Laboratory, Pacific Biomedical Research Center, University of Hawaii, 41 Ahui Street, Honolulu, HI 96813, USA

## Abstract

**Background:**

The transcription factors of the LSF/Grainyhead (GRH) family are characterized by the possession of a distinctive DNA-binding domain that bears no clear relationship to other known DNA-binding domains, with the possible exception of the p53 core domain. In triploblastic animals, the LSF and GRH subfamilies have diverged extensively with respect to their biological roles, general expression patterns, and mechanism of DNA binding. For example, *Grainyhead *(GRH) homologs are expressed primarily in the epidermis, and they appear to play an ancient role in maintaining the epidermal barrier. By contrast, LSF homologs are more widely expressed, and they regulate general cellular functions such as cell cycle progression and survival in addition to cell-lineage specific gene expression.

**Results:**

To illuminate the early evolution of this family and reconstruct the functional divergence of LSF and GRH, we compared homologs from 18 phylogenetically diverse taxa, including four basal animals (*Nematostella vectensis*, *Vallicula multiformis*, *Trichoplax adhaerens*, and *Amphimedon queenslandica*), a choanoflagellate (*Monosiga brevicollis*) and several fungi. Phylogenetic and bioinformatic analyses of these sequences indicate that (1) the LSF/GRH gene family originated prior to the animal-fungal divergence, and (2) the functional diversification of the LSF and GRH subfamilies occurred prior to the divergence between sponges and eumetazoans. Aspects of the domain architecture of LSF/GRH proteins are well conserved between fungi, choanoflagellates, and metazoans, though within the Metazoa, the LSF and GRH families are clearly distinct. We failed to identify a convincing LSF/GRH homolog in the sequenced genomes of the algae *Volvox carteri *and *Chlamydomonas reinhardtii *or the amoebozoan *Dictyostelium purpureum*. Interestingly, the ancestral GRH locus has become split into two separate loci in the sea anemone *Nematostella*, with one locus encoding a DNA binding domain and the other locus encoding the dimerization domain.

**Conclusions:**

In metazoans, LSF and GRH proteins play a number of roles that are essential to achieving and maintaining multicellularity. It is now clear that this protein family already existed in the unicellular ancestor of animals, choanoflagellates, and fungi. However, the diversification of distinct LSF and GRH subfamilies appears to be a metazoan invention. Given the conserved role of GRH in maintaining epithelial integrity in vertebrates, insects, and nematodes, it is noteworthy that the evolutionary origin of Grh appears roughly coincident with the evolutionary origin of the epithelium.

## Background

In triploblastic animals, LSF/Grainyhead (GRH) transcription factors perform a number of functions essential to both development and homeostasis. They are involved in regulation of the cell cycle, cell division, and cellular differentiation in a range of developmental and non-developmental contexts [1-14].

The LSF/Grainyhead family is split into the LSF/CP2 subfamily and the Grainyhead (GRH) subfamily, which can be distinguished by their distinctive oligomerization domains and differences in their oligomerization behavior [10,15,16]. GRH binds to DNA as a dimer, whereas LSF binds as a tetramer [17,18]. The DNA binding regions in both protein subfamilies show a large amount of conservation [17,18], but each has distinct transcriptional targets. GRH binds to the DNA sequence: (A/T)C(A/C/T)(G/T)GTT(C/G/T), whereas LSF binds to a direct repeat with the consensus sequence of N(C/G/T)N(C/G/T)(C/G)N(C/T)N(C/G/T)NN(C/G/T)(C/G/T)N(A/C/G)N [15,16,18,19]. LSF proteins can also be distinguished from GRH by the possession of a sterile alpha motif (SAM) [20]. Members of both LSF and GRH subfamilies were previously identified in vertebrates, arthropods, and nematodes, so the origin of the family and the diversification into subfamilies is known to predate the evolutionary split between protostomes and deuterostomes [15]. Recently, a common origin for the LSF/GRH family and the p53 family has been proposed based on similarities in the folding of their DNA-binding domains [20].

The differences in the molecular functions of LSF and GRH are accompanied by important differences in their biological roles. In both vertebrates and protostome invertebrates, GRH proteins are involved in the development and maintenance of epithelial integrity [21]. For example, in mice, *grh *is required during embryogenesis where it is expressed exclusively in the developing ectodermal epithelium [22]. Furthermore, embryonic mice lacking *grhl-3 *exhibit insufficient wound repair and abnormal skin barrier formation leading to excessive postnatal water loss. The water loss is associated with reduced expression of the gene encoding *TGase1*, an enzyme that promotes cross-linking of parts of the stratum corneum, thus preventing the movement of water and solutes [22]. Likewise, in *Xenopus*, a *Grh*-like gene (*Xgrh1*) has been implicated in the development of the epidermis [13]. One of its primary targets is epidermal keratin. In morpholino studies, knockdown of *Xgrhl *led to loss of surface structures and pigmentation as well as neck and eye defects associated with epidermal instability [13]. In *Drosophila*, GRH plays a critical role in epithelial integrity that is analogous to and perhaps homologous with the role played in vertebrates--GRH maintains the tension of the *Drosophila *cuticle, and it induces cuticle development and cuticle repair following injury [23,24]. Similarly, the CeGrh1 protein of *C. elegans *appears to be required for proper cuticle formation during development, as its knockdown leads to soft, malformed cuticles and embryonic lethality [15].

In addition to its widely conserved role in maintaining epidermal integrity, *Grh *is also involved in the specification and development of the CNS in both *Drosophila *and mice [9,25]. Additionally, in mice, *Grh *mutants exhibit defects of the salivary and kidney ducts and eyelid closure [26-28], and in humans, a single nucleotide polymorphism found in GRHL2 is associated with age-related hearing impairment [29].

The biological roles of LSF are diverse and they have clearly diverged from those of GRH, at least in mammals, where the function of LSF has been well characterized. LSF is ubiquitously expressed [30]. It appears to play a role in liver function, eye development, erythropoesis, neural and immune function, regulation of the cell cycle progression, and cell survival [8,16,31-42].

When the ancestors of the LSF and GRH subfamilies first originated via a gene duplication event from their common ancestor, they would presumably have had identical or largely overlapping functions. However, at least in extant mammals, LSF and GRH have diverged extensively with respect to their biological roles. The basis for this functional diversification is not clear. The common ancestral functional repertoire of LSF and GRH may have become "subfunctionalized" in the two descendants [43]. Alternatively or in concert, LSF and GRH may have independently acquired novel functions since their split from a common ancestral gene ("neofunctionalization") [43,44].

If we wish to reconstruct the initial functional diversification of LSF and GRH, it is necessary to identify the ancestor in which the original gene duplication occurred. This may permit us to infer the functional repertoire of the LSF/GRH ancestor, and to compare this ancestral condition with the function of LSF and GRH in a phylogenetic progression of extant taxa. By comparing vertebrates, arthropods, and nematodes, Venkatesan and co-workers previously showed that the origin of distinct LSF and GRH subfamilies predated the diversification of triploblasts into distinct protostome and deuterostome lineages [15]. With the recent availability of sequenced genomes from several basal metazoans, a choanoflagellate, and more distantly related fungal outgroups, we can track the evolution of the LSF/GRH family into the much more distant past. In this study, we report the identification of LSF/GRH family members in 24 previously unreported species. Through a combination of genome prospecting and phylogenetic analysis, we show that the original gene duplication that produced the LSF and GRH subfamilies occurred prior to the evolutionary radiation of basal animal lineages (e.g., Bilateria, Cnidaria, Ctenophora, Porifera, and Placozoa). Interestingly, the GRH protein of the sea anemone *Nematostella vectensis*, a representative cnidarian, appears to have split into two distinct loci. We also identify six protein motifs that are widely shared between the LSF and GRH subfamilies of metazoans, all of which can be traced to the common ancestor of metazoans and fungi. In addition, there is a single motif that appears unique to the LSF subfamily.

## Results

### Identification of putative LSF/GRH homologs in animals, choanoflagellates, and fungi

BLAST searches identified putative LSF and GRH orthologs in eleven non-mammalian animals (Table 1) including three chordates (*Branchiostoma floridae*, *Ciona intestinalis*, *Fugu rubripes*), three arthropods (*Anopheles gambiae*, *Daphnia pulex*, and *Drosophila melanogaster*), an annelid (*Capitella *spp.), a mollusc (*Lottia gigantea*), a cnidarian (*Nematostella vectensis*), and a sponge (*Amphimedon queenslandica*). We also identified a strong match to human GRHL2 in the ctenophore (*Vallicula multiformis) *and the placozoan *(Trichoplax adhaerens)*. We were not able to identify putative LSF orthologs in either the ctenophore or the placozoan.

**Table 1:** LSF/GRH sequences identified by BLAST searches.

Species		Protein	^3^E-value	Accession
**Choanoflagellata**				

^1,2^*Monosiga brevicollis	Mob	LSF-like	3e-10	jgi| Monbr1| 29664| fgenesh2_pg.scaffold_37000034

**Fungi/Ascomycota**				

^1,2^*Aspergillus niger	Asn	LSF-like1	8e-18	jgi| Aspni1| 174592| e_gw1.2.1262.1

^1,2^*Aspergillus niger	Asn	LSF-like2	1e-6	jgi| Aspni1| 206941| estExt_GeneWisePlus.C_21219

^2^*Mycosphaerella fijiensis	Myf	LSF-like	7e-39	jgi| Mycfi1| 87221| estExt_fgenesh1_pg.C_60133

^2^*Mycosphaerella graminicola	Myg	LSF-like	9e-8	jgi| Mycgr3| 76636| estExt_Genewise1Plus.C_chr_110062

^2^*Trichoderma virens	Trv	LSF-like	2e-5	jgi| Trive1| 37466| e_gw1.6.78.1

**Fungi/Basidiomycota**				

*Phanerochaete chrysosporium	Phc		.058	jgi| Phchr1| 428| fgenesh1_pg.C_scaffold_1000428

*Sporobolomyces roseus	Spr		.014	jgi| Sporo1| 4513| gw1.5.455.1

**Fungi/Zygomycota**				

^1,2^*Phycomyces blakesleeanus	Phb	LSF-like1	2e-32	jgi| Phybl1| 75515| estExt_fgeneshPB_pg.C_10328

^1,2^*Phycomyces blakesleeanus	Phb	LSF-like2	8e-14	jgi| Phybl1| 63864| fgeneshPB_pg.8__95

**Metazoa/Annelida**				

^1,2^*Capitella species	Cap	GRH	7e-41	jgi| Capca1| 198092| fgenesh1_pg.C_scaffold_3000030

^1,2^*Capitella species	Cap	LSF	e-119	>jgi| Capca1| 222821| estExt_fgenesh1_pg.C_600043

**Metazoa/Arthropoda**				

*Anopheles gambiae*	Ang	GRH		ref| XP_308698.4|

*Anopheles gambiae*	Ang	LSF		ref| XP_315571.4|

^1,2^*Daphnia pulex	Dap	GRH	8e-43	jgi| Dappu1| 64332| e_gw1.171.21.1

^1,2^*Daphnia pulex	Dap	LSF	e-127	jgi| Dappu1| 192185| estExt_Genewise1Plus.C_50175

^1,2^*Drosophilia melanogaster*	Drm	GRH	**	ref| NP_476842.2|

^1,2^*Drosophilia melanogaster	Drm	Gemini	**	ref| NP_610556.1|

**Metazoa/Chordata**				

^1,2^*Branchiostoma floridae	Brf	GRH	e-129	jgi| Brafl1| 106909| fgenesh2_pg.scaffold_554000005

^1,2^*Branchiostoma floridae	Brf	LSF	e-146	jgi| Brafl1| 129980| estExt_fgenesh2_pg.C_4020027

^1,2^*Ciona intestinalis	Cii	GRH	e-107	jgi| Cioin2| 262638| gw1.01q.820.1

^1,2^*Ciona intestinalis	Cii	LSF	e-141	jgi| Cioin2| 262956| gw1.03q.546.1

*Fugu rubripes	Fur	GRH	0	jgi| Takru4| 570042| e_gw2.138.33.1

*Fugu rubripes	Fur	LSF	0	jgi| Takru4| 710919| fgh5_pm.C_scaffold_60000044

*Homo sapiens*	Hos	CP2		ref| NP_005644.2|

*Homo sapiens*	Hos	CP2like3		ref| NP_079191.2|

*Homo sapiens*	Hos	GRHL1		gb| AAH67519.1|

^1,2^*Homo sapiens*	Hos	GRHL2		gb| AAH69633.1|

^1,2^*Homo sapiens*	Hos	LBP1a		gb| AAH47235.1|

*Homo sapiens*	Hos	LBP9		ref| NP_055368.1|

*Homo sapiens*	Hos	LBP32		gb| AAF32276.1| AF198489_1

*Homo sapiens*	Hos	SOM1		ref| NP_067003.2|

*Mus musculus*	Mum	BOM		AAM22619

*Mus musculus*	Mum	CP2		ref| NP_258437.1|

*Mus musculus*	Mum	CRTR-1		NP_076244

*Mus musculus*	Mum	NF2d9		AAC52244

**Metazoa/Cnidaria**				

^1,2^*Nematostella vectensis	Nev	GRH1	2e-49	jgi| Nemve1| 95157| e_gw.38.94.1

^1,2^*Nematostella vectensis	Nev	LSF	3e-88	jgi| Nemve1| 189242| estExt_GenewiseH_1.C_1380090

**Metazoa/Ctenophora**				

^1,2^* *Vallicula multiformis*	Mnl	GRH		EST

**Metazoa/Mollusca**				

^1,2^*Lottia gigantea	Log	GRH	3e-52	jgi| Lotgi1| 157385| fgenesh2_pg.C_sca_14000008

^1,2^*Lottia gigantea	Log	LSF	e-122	jgi| Lotgi1| 166840| fgenesh2_pg.C_sca_61000088

**Metazoa/Placozoa**				

^2^*Trichoplax adhaerens	Tra	GRH-like	4e-20	jgi| Triad1| 25702| e_gw1.5.1214.1

**Metazoa/Porifera**				

^1,2^*Amphimedon queenslandica	Amq	GRH	7e-55	Not Available

^1,2^*Amphimedon queenslandica	Amq	LSF	4e-49	Not Available

**Plantae**				

*Selaginella moellendorffii	Sem		.019	jgi| Selmo1| 411196| fgenesh2_pg.C_scaffold_14000249

*Chlamydomonas	Chl		.042	jgi| Chlre3| 194996| estExt_fgenesh2_pg.C_840034

^1^Sequence included in MEME analysis; ^2^Sequence included in phylogenetic analysis; ^3 ^Expectation value of match to human query (either GRHL2^4 ^or LBP1a^5^) observed during BLAST searches. Asterisks identify newly acquired sequences.

The cnidarian *Nematostella *is unusual in that its GRH homolog appears to be split between two loci. Nev-GRH1, which had been reported previously [45], emerged as a strong match to the entire human GRHL2 protein. However, as Nev-GRH1 appears to be truncated relative to the human protein, we conducted a separate BLAST search using only the carboxy terminal region of the human protein as a query sequence. Nev-GRH2, which was identified in this second BLAST search, is a strong match to the carboxy terminal portion of the human GRHL2 protein.

Among choanoflagellates and fungi, we were also able to identify members of the LSF/GRH family, but clear evidence for distinct LSF and GRH family members was less compelling. The sequenced genome of the choanoflagellate *Monosiga brevicollis *appears to encode only a single LSF/GRH related gene. Likewise, we could identify only a single LSF/GRH homolog in the genomes of four fungi (*Mycosphaerella fijiensis*, *Mycosphaerella graminicola*, *Phanerochaete chrysosporium *and *Trichoderma virens*). We did identify two LSF/GRH-related sequences in *Aspergillus niger *(phylum Ascomycota) and *Phycomyces brevicollis *(phylum Zygomycota), but in both cases, the two sequences appeared most similar to each other, suggesting that they might have resulted from lineage-specific gene duplications.

In members of the kingdom Plantae, evidence of LSF/GRH family members was far more tenuous. Using a less stringent E-value cut off (e-1), we identified two proteins with limited resemblance to LSF/GRH in *Selaginella moellendorffii*, a lycophyte. In addition, we also identified a protein with similarity to LSF in the green algae *Chlamydomonas reinhardtii*. Using this *Chlamydomonas *sequence to query the genome of *Volvox carteri*, we identified the corresponding gene in this alga.

### Protein motif identification

**Figure 1:**
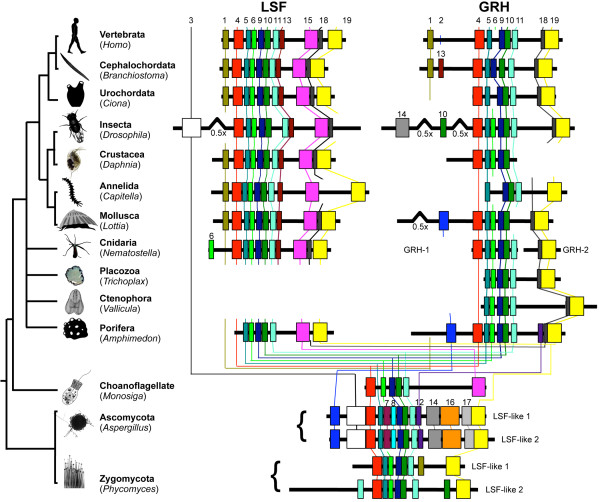
**Motif architecture of LSF and GRH proteins from 10 metazoan taxa, a choanoflagellate, and two fungi**. Conserved motifs were identified using MEME, as described in the methods. Motifs (colored boxes) and inter-motif regions (thick black lines) were drawn to scale except for certain lengthy inter-motif regions, which were truncated by 50% (0.5×). Thin colored lines highlight motif conservation between proteins. The relative relationships among taxa depicted here reflect a general consensus among molecular phylogenetic analyses [62-68], although there continues to be controversy surrounding key elements of the phylogeny including the placement of ctenophores [69] and the monophyly of the triploblasts [70-72].

**Figure 2:**
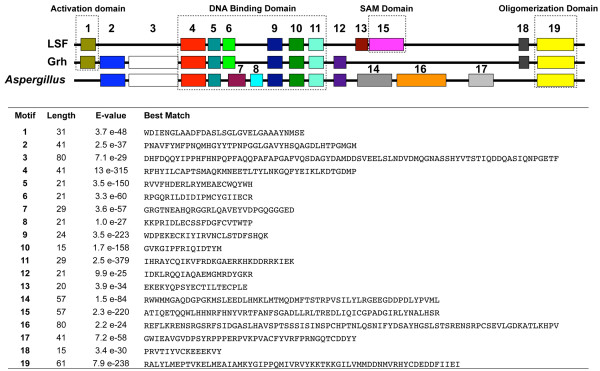
**Top-scoring motif sequences and consensus motif architecture**. Metazoan LSF proteins, metazoan GRH proteins, and fungal proteins can be distinguished by their consensus motif architectures (top). The consensus diagrams include all motifs that were found in at least one member of the respective group (Fig. 1). The best matches for each sequence motif identified by MEME are shown below the diagrams. The correspondence between these conserved motifs and known functional domains are indicated by boxes.

MEME analysis (Additional file 1) reveals extensive conservation in motif architecture within and between the LSF and GRH proteins of animals; it also reveals extensive conservation between these animal proteins and the LSF/GRH-related proteins of the choanoflagellate and the fungi (Figure 1). Overall, the MEME analysis identified 19 motifs that exhibit significant conservation between two or more sequences (Figure 2). Six motifs (4, 5, 6, 9, 10 and 11) are almost universally conserved among animal, choanoflagellate, and fungal sequences. Several of these motifs either correspond to previously identified functional domains, or they reside within such domains. Motif 1 corresponds to the activation domain [3,16,46]. Motifs 4, 5, 6, 9, 10 and 11 reside within the DNA binding domain [18,20,47]. Motif 15 corresponds to the SAM domain, and motifs 18 and 19 correspond to the dimerization domain. Two adjacent motifs (13 and 15) are well conserved among LSF proteins. While motif 15 was also identified in the choanoflagellate protein, the co-occurrence of motifs 13 and 15 appears characteristic of the LSF subfamily, with the exception of the sponge LSF sequence that did not exhibit a significant match to motif 13.

The motif analysis reveals strong similarities between the pairs of sequences identified in each of the two fungal species. The two proteins from the ascomycote fungus, *Aspergillus*, are nearly identical to each with respect to motif architecture, and they can be distinguished from other sequences by the possession of motifs 14, 16, and 17. Likewise, the two sequences from the zygomycote fungus, *Phycomyces*, are most similar to each other with respect to the arrangement of conserved motifs.

The motif analysis also supports the conclusion that the GRH locus of the cnidarian *Nematostella *has experienced a split. Nev-GRH1 encompasses six conserved motifs (4, 5, 6, 9, 10, 11), and these motifs occupy the same relative positions as in the GRH proteins of fruit fly and sponge. Nev-GRH2 encompasses conserved motifs 18 and 19, which occupy the same relative position in most other metazoan GRH sequences.

### Phylogenetic analysis

**Figure 3:**
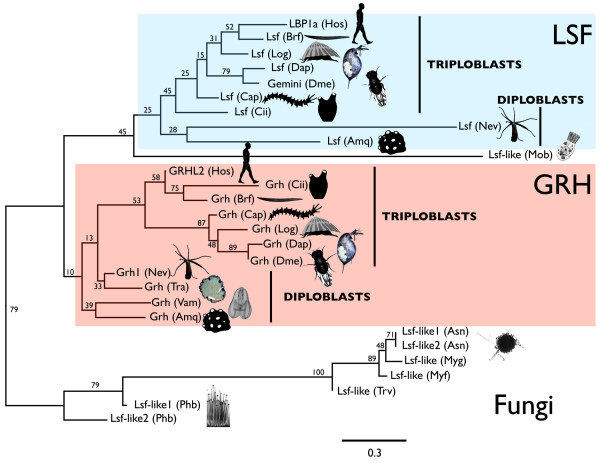
**Phylogeny of LSF and GRH proteins**. The tree shown is based on a neighbor-joining analysis of the amino acids in the gap free alignment. Numbers at nodes represent bootstrap support. The tree is drawn as though rooted between the metazoan sequences and the fungal sequences. Branch length is shown in terms of expected number of substitutions per residue (bar at lower right).

All phylogenetic analyses that we performed can be rooted so that the fungal sequences and the metazoan sequences form mutually exclusive monophyletic groups (Figure 3; Additional file 2). On the neighbor-joining tree (Figure 3), the metazoan clade can be further subdivided into putative LSF and GRH clades. Within the LSF clade, the triploblastic animals form a monophyletic group to the exclusion of two diploblastic animals (*Nematostella *and *Amphimedon*). Similarly, within the GRH clade, the triploblastic animals form a monophyletic group to the exclusion of four diploblastic animals (*Nematostella*, *Amphimedon*, *Trichoplax*, and *Vallicula*), implying that both LSF and GRH subfamilies had originated prior to the evolutionary split between diploblasts and triploblasts. The single *Trichoplax *sequence groups within the GRH clade. Though the bootstrap support for this grouping is low, along with the motif analysis, this suggests that the *Trichoplax *sequence may be a true GRH ortholog (implying that the LSF ortholog of *Trichoplax *has either been lost or we failed to find it). The single *Monosiga *sequence appears at the base of the LSF clade, suggesting that it might be a true LSF ortholog (which would imply that LSF and GRH diverged before the split between animals and choanoflagellates). The single *Vallicula *sequence groups with GRH sequences of other diploblastic animals.

The maximum-likelihood analysis (Additional file 2) supports most of the major divisions that appear on the neighbor-joining tree. The animal sequences and fungal sequences comprise discrete subtrees. The LSF sequences form a putative clade, and within this clade, the LSF sequences of triploblasts cluster together to the exclusion of LSF sequences from diploblasts. Likewise, the GRH sequences of triploblasts also group together. However, the putative GRH sequences of diploblastic animals do not form a monophyletic group with the GRH sequences of triploblasts as they do on the neighbor-joining tree. Instead, the sponge and ctenophore sequences appear more closely related to the LSF clade, while the precise position of the anemone and placozoan GRH sequences is not resolved.

On both the neighbor-joining tree and the maximum-likelihood tree, bootstrap support for individual nodes is generally low because the analyses are based on a small number of highly conserved residues. However, both phylogenies are consistent with divisions between animal and fungal sequences and between LSF and GRH sequences, the same divisions that are implied by the motif analysis.

### Nev-GRH1 and Nev-GRH2

**Figure 4:**
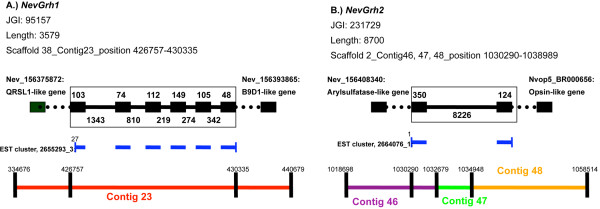
**Mapping of *Nev-Grh1 *and *Nev-Grh2 *ESTs to separate loci**. The *NevGrh1 *and *NevGrh2 *loci (enclosed in boxes) are flanked by distinct genes and are found on distinct, non-overlapping genomic scaffolds. Exons are indicated by black boxes, and introns are represented by solid black lines. Dotted lines represent the intergenic sequence leading to the nearest flanking genes. Flanking genes are named by species and NCBI number. The EST contigs for each locus are represented as thick blue lines beneath the exons that encode them. Figure is not to scale.

The sea anemone, *Nematostella vectensis*, is unique in that the GRH locus has been split in two, with Nev-Grh1 encoding primarily the DNA-binding domain and Nev-Grh2 encoding primarily the dimerization domain. In the current draft assembly of the genome, Nev-Grh1 maps to scaffold 2, and Nev-Grh1 maps to scaffold 38 (Joint Genome Institute, *Nematostella vectensis *v1.0; Figure 4). Nev-Grh1 is flanked by a QRSL1 like gene and a B9D1-like gene. Nev-Grh2 is flanked by an arylsulfatase-like gene and an opsin-like gene. Even if these two scaffolds reside on the same chromosome, based on the location of each gene within its respective scaffold, the two loci must be separated by no less than 580 kilobases of intervening sequence. Both of the Grh loci are represented by multiple ESTs (NevGRH1, EST cluster: 2655293_3; NevGRH2, EST cluster: 2664076_1), and none of the individual ESTs overlap (thus, there is no evidence for trans-splicing).

### Potential homologs in plants?

Given that the origin of the LSF/GRH family predates the divergence of animals and fungi, we searched for LSF and GRH homologs in amoebozoans and plants to see if this gene family might predate the origin of opisthokonts. Plant genomes and amoebozoan genomes do not appear to encode any proteins with extensive similarity to the LSF/GRH proteins of animals and fungi. In tblastn searches of assembled genomes at the JGI Genome Portal [48] using a permissive E value cut-off (e-1), the lycophyte, *Selaginella moellendorffii*, yielded a hit for GRH (E value 0.07), the alga *Chlamydomonas *yielded a hit for LSF (E value 0.07), and the amoebozoan *Dictyostelium purpureum *yielded a hit for LSF (E value 0.04; Additional file 3). When the top hit from *Selaginella *and *Dictyostelium *were BLASTed back against the human genome, the search yielded no significant hits.

## Discussion

### Evolutionary origins of the LSF/GRH family and subfamilies

Prior to the present study, members of the LSF/GRH family had been reported from a number of triploblastic animals but not from diploblastic animals, choanoflagellates, or fungi. We recovered clear LSF and GRH orthologs from two diploblastic animals (sea anemone and sponge) revealing that the evolutionary divergence between these two subfamilies must have predated the diploblast-triploblast split. Furthermore, fungi possess clear LSF/GRH homologs, although the fungal sequences cannot be assigned to either the LSF or GRH subfamilies. Therefore, while the family clearly originated prior to the metazoan-fungal divergence, the diversification of subfamilies occurred more recently, perhaps in an ancient animal lineage.

### Nev-GRH1 and Nev-GRH2

The sea anemone, *Nematostella vectensis*, is the only species where the sequences encoding the ancestral GRH protein are known to be split between two loci. As sponges, ctenophores, and triploblasts exhibit full-length GRH proteins, this condition must be derived in the sea anemone. The splitting of the ancestral Grh locus in *Nematostella *must have profound consequences for the regulation and function of GRH. In other animals, GRH binds DNA targets as a dimer. However, in *Nematostella*, the DNA-binding domain and the oligomerization reside on different proteins. Perhaps Nev-GRH1 is able to interact with the DNA singly, or perhaps a partnership with Nev-GRH2 allows it to form the equivalent of a GRH-dimer on DNA, reminiscent of other GRH proteins. This latter possibility implies that Nev-GRH1 and Nev-GRH2 will be co-expressed in the same cells. This will need to be confirmed experimentally. Interestingly, a comparable split seems to have occurred in the NF-κB gene of this species, with distinct loci encoding different functional domains of the ancestral protein [49].

### Identification of LSF/GRH Homologs in Fungi

Convincing matches to human LSF and/or GRH query sequences were found in the genomes of representative ascomycote, basiomycote and zygomycote fungi (Table 1). The phylum Basiomycota is the sister group to the phylum Ascomycota, with the Zygomycota being more distantly related [50], and the phylogenetic analysis we performed grouped the LSF-like proteins of the ascomycotes *Aspergillus*, *Mycosphaerella*, and *Trichoderma *to the exclusion of the LSF-like proteins from the zygomycote *Phycomyces*. In the MEME analysis, the two LSF/GRH proteins identified in the zygomycote *Phycomyces *were found to possess all of the conserved motifs that were identified within the DNA-binding domain of animals (motifs 4, 5, 6, 9, 10, and 11). The two LSF/GRH proteins of the ascomycote, *Aspergillus*, also possess motifs 4, 5, 9, 10, and 11, but in place of motif 6, these proteins share motifs 7 and 8, which are unique to this fungus. All four fungal sequences subjected to the MEME analysis were found to contain motif 19, which corresponds to the dimerization domain. Given the strong conservation of motifs between fungi and animals in the DNA-binding and dimerization domains, we hypothesize that the molecular function of these fungal proteins will be very similar to their animal homologs, *i.e*., they are transcription factors that will bind DNA targets, most likely as dimers (like GRH). However, if the novel fungal-specific motifs functionally replace the SAM domain, which is likely to represent the second protein-protein interaction domain in LSF subfamily members, they might instead bind DNA as tetramers (like LSF).

### Insights into the hypothesized ancestral role of GRH from basal animals

Because GRH plays a comparable role in the maintenance and repair of the surface epithelium in mouse [22], clawed frog [13], fruit fly [23], and soil nematode [15], it has been hypothesized that this role is homologous among triploblastic bilaterians [21,24]. Given that the shared possession of an epithelium is thought to be homologous across the Metazoa, it is possible that the functional evolution of GRH is connected to the origin and early evolution of the epithelium. The presence of an epithelial boundary is a plesiomorphic character of triploblastic animals, and therefore, we cannot explore the early evolution of animal epithelia using only triploblastic model systems. The identification of clear GRH homologs in cnidarians, ctenophores, and sponges, and the apparent absence of a true *Grh *gene in the choanoflagellate *Monosiga *suggests the origin of *Grh *may be coincident with the origin of the metazoan epithelium. Historically, sponges have been said to lack an epithelium, but more recently, the identification of a genuine basement membrane in homoscleromorph sponges removes this distinction between poriferans and other metazoans [51]. If the role of *Grh *in maintaining epithelial integrity dates to the origin of the epithelium, then *Grh *should be expressed in the epidermal epithelium of cnidarians, ctenophores and sponges. Furthermore, *Grh *should regulate proteins involved in epithelial differentiation and maintenance, although the exact targets of *Grh *transcriptional regulation may vary among basal animals as they vary among triploblasts. Additionally, we may expect that *Grh *will be upregulated in response to injury, while knockdown of *Grh *expression may undermine epithelial integrity and inhibit wound healing. All of these questions are amenable to testing in one or more basal model systems.

## Conclusions

The LSF/GRH family had already originated by the time of the opisthokont ancestor, and the overall domain architecture of LSF/GRH proteins has been largely conserved in extant fungi, animals, and choanoflagellates. The LSF subfamily had diverged from the GRH family prior to the divergence of sponges, cnidarians, and triploblastic animals. Consistent differences in domain architecture distinguish the LSF and GRH proteins of both diploblastic and triploblastic animals, suggesting that the functional divergence between these proteins had been established prior to the evolutionary divergence between diploblasts and triploblasts. The sea anemone *Nematostella *appears unique in that the DNA-binding domain and the dimerization domain of the ancestral GRH protein are now encoded on two separate loci.

## Methods

### Identification of LSF/Grainyhead family members in outgroup taxa

The human proteins LSF [NP_005644.2] and GRHL2 [AAH69633.1] were used to query online genomic databases (Joint Genome Institute Eukaryotic Genomes and NCBI) for LSF-like and GRH-like proteins respectively using BlastP. The following search settings were employed: gap opening penalty = 11; gap extension penalty = 1. Potential homologs that matched one of the query sequences with an expectation score < e-1 were used to query the human genome (using BLASTp) to determine if their top human match was to the original human query sequence (LSF or GRHL2). Sequences were kept for phylogenetic and protein motif identification only if they met this criterion.

### Protein motif identification

To identify conserved protein motifs, LSF/GRH proteins were evaluated using MEME (Multiple Expectation Maximization for Motif Elicitation; http://meme.nbcr.net; ([52]; Additional file 1). LSF/GRH family members were chosen to represent ten metazoan phyla, the choanoflagellate *Monosiga brevicollis*, an ascomycote fungus (*Aspergillus*) and a zygomycote fungus (*Phycomyces*; Table 1). The following settings were used in the motif search: maximum number of motifs = 20; occurrences of a single motif = any number; minimum length of a motif = 3 amino acids; maximum length of a motif = 300.

### Multisequence Alignment

Twenty-eight of the twenty-nine LSF/GRH protein sequences included in the MEME analysis were aligned in preparation for phylogenetic analysis (Additional file 4). The GRH2 protein of *Nematostella vectensis *was excluded from the alignment because it is substantially truncated relative to the full-length LSF and GRH proteins of other animals. Since motif 4 was identified near the amino terminal of all but three of the proteins, and motif 19 was identified near the carboxy terminal of all but one of the proteins (Figure 1), these motifs were used to bracket the alignment. To ensure that the motifs identified by MEME were maintained in register, the motifs themselves were manually aligned. Then, the regions between conserved motifs were multiply aligned using the Clustal alignment tool found in the application MEGA [53]. The following settings were specified: protein weight matrix = Gonnet, gap opening penalty = 10; gap extension penalty = 0.2. The resulting alignment spans 2045 characters. All positions in the alignment containing gaps were deleted to produce a gap-free alignment comprising 44 characters (Additional file 4).

### Phylogenetic Analysis

Phylogenetic relationships among taxa were inferred from both the gap-free alignment and the full alignment using neighbor-joining [54] and maximum-likelihood [55]. All 44 residues in the gap-free alignment derive from motifs 9-11, which are part of the DNA-binding domain (Additional file 2). First, eighty alternate models of the amino acid substitution process were compared using the program ProtTest 1.3 [56]. The substitution process was optimized along with the tree topology and branch lengths. For both the full alignment and the gap-free alignment, the empirically determined JTT substitution matrix [57] outperformed other substitution matrices, and incorporating rate variation among sites significantly improved the model (the shape coefficient of the Gamma distribution, α = .837; the coefficient of rate variation among sites = 1/α^1/2 ^= 1.093). The JTT matrix with gamma-distributed rate variation among sites was specified in subsequent phylogenetic analyses.

For the neighbor joining analysis, pairwise distances between proteins were calculated using the Prodist program, and the tree topology was determined using the Neighbor program, both in the Phylip package (v. 3.6; [58]). Maximum-likelihood analysis was performed using RAxML (v 7.0.3; [59]) as implemented on the CIPRES Portal (v. 2.0; [60]). In both the neighbor-joining analysis and the maximum-likelihood analysis, support for specific clades was assessed using the bootstrap [61]: 1,000 replicates of the bootstrap were performed for the neighbor-joining analysis, and 100 replicates were performed for the maximum-likelihood analysis.

## Authors' contributions

NTK performed the sequence acquisition and collaborated on the phylogenetic analysis and protein motif analysis. UH contributed to the data analysis of protein motifs. TQD contributed the *V. multiformis *sequence. MQM contributed to data interpretation. LK contributed to the conception of the project. JRF contributed to the conception of the project, data analysis and interpretation, phylogenetic analysis, and protein motif analysis. All authors contributed to writing the manuscript and read and approved the final manuscript.

## Supplementary Material

Additional file 1 Output from the motif discovery program MEME. Click here for file

Additional file 2 **The tree shown is based on a maximum-likelihood analysis of the amino acids in the gap free alignment using the program RAxML**[73]. Numbers at nodes represent bootstrap support. The tree is drawn as though rooted between the metazoan sequences and the fungal sequences. Branch length is shown in terms of expected number of substitutions per residue (bar at lower left). Click here for file

Additional file 3 **The assembled genomes of***Chlamydomonas reinhardtii*, *Dictyostelium purpureum*, **and ***Selaginella moellendorffii *(**housed at the JGI Genome Portal **[48])** were queried with a human LSF sequence (>gi|21361278|ref|NP_005644.2| transcription factor CP2) and GRH sequence (>gi|46854865|gb|AAH69633.1| GRHL2 protein).** A permissive E value cut-off was specified (e-1). *Dictyostelium *and *Chlamydomonas *each returned a single hit for LSF, and *Selaginella *returned a single hit for GRH. Click here for file

Additional file 4 All positions in the full alignment containing gaps were deleted to produce this gap-free alignment comprising 44 characters. Click here for file
