# Supplementary figures and images for: The evolutionary diversification of LSF and Grainyhead transcription factors preceded the radiation of basal animal lineages
Source: BMC Evol Biol. 2010 Apr 18;10:101. doi: 10.1186/1471-2148-10-101 (PMC2873413; doi:10.1186/1471-2148-10-101)

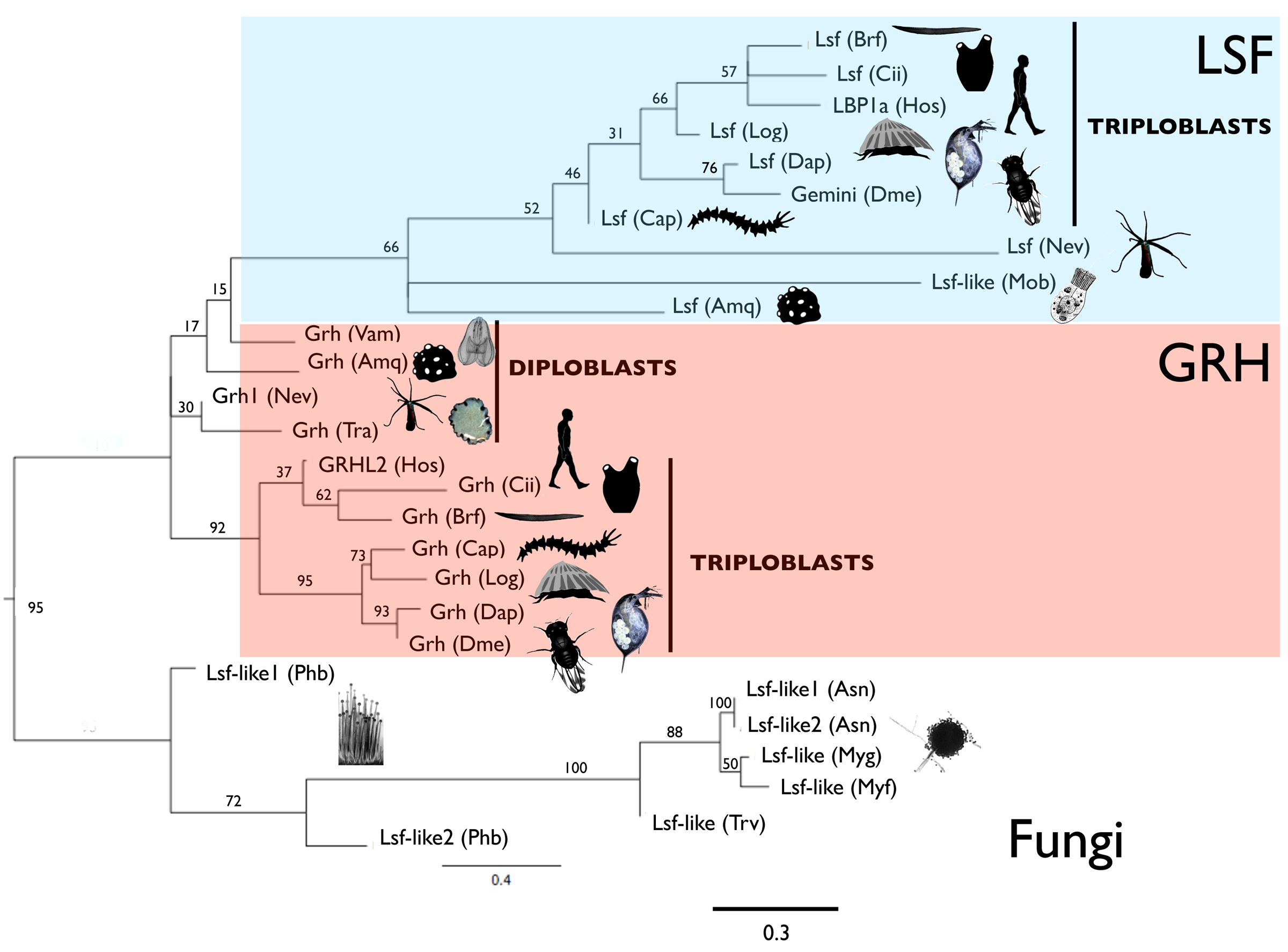

Supplement: Additional file 2 — The tree shown is based on a maximum-likelihood analysis of the amino acids in the gap free alignment using the program RAxML[73]. Numbers at nodes represent bootstrap support. The tree is drawn as though rooted between the metazoan sequences and the fungal sequences. Branch length is shown in terms of expected number of substitutions per residue (bar at lower left). [file 1471-2148-10-101-S2.TIFF]
