# Supplementary material for: The evolutionary diversification of LSF and Grainyhead transcription factors preceded the radiation of basal animal lineages
Source: BMC Evol Biol. 2010 Apr 18;10:101. doi: 10.1186/1471-2148-10-101 (PMC2873413; doi:10.1186/1471-2148-10-101)
Supplement: Additional file 3 — The assembled genomes ofChlamydomonas reinhardtii, Dictyostelium purpureum, and Selaginella moellendorffii (housed at the JGI Genome Portal [48]) were queried with a human LSF sequence (>gi|21361278|ref|NP_005644.2| transcription factor CP2) and GRH sequence (>gi|46854865|gb|AAH69633.1| GRHL2 protein). A permissive E value cut-off was specified (e-1). Dictyostelium and Chlamydomonas each returned a single hit for LSF, and Selaginella returned a single hit for GRH. [file 1471-2148-10-101-S3.PDF]

**Defline**	query
	query
**Length**	502
**Scaffold**	scaffold_27 (256895 bp) : 137529:247506 (109978 bp)
**Others**	Other scaffolds hit by this sequence
**Browser**	View on browser

xstart: 1 **xend:** 502
ystart: 137529 **yend:** 247506

Rescale Reset

Color Key for BLAST Alignment Scores:
< 40 | 40-50 | 50-80 | 80-200 | >=200

Linear Graphic

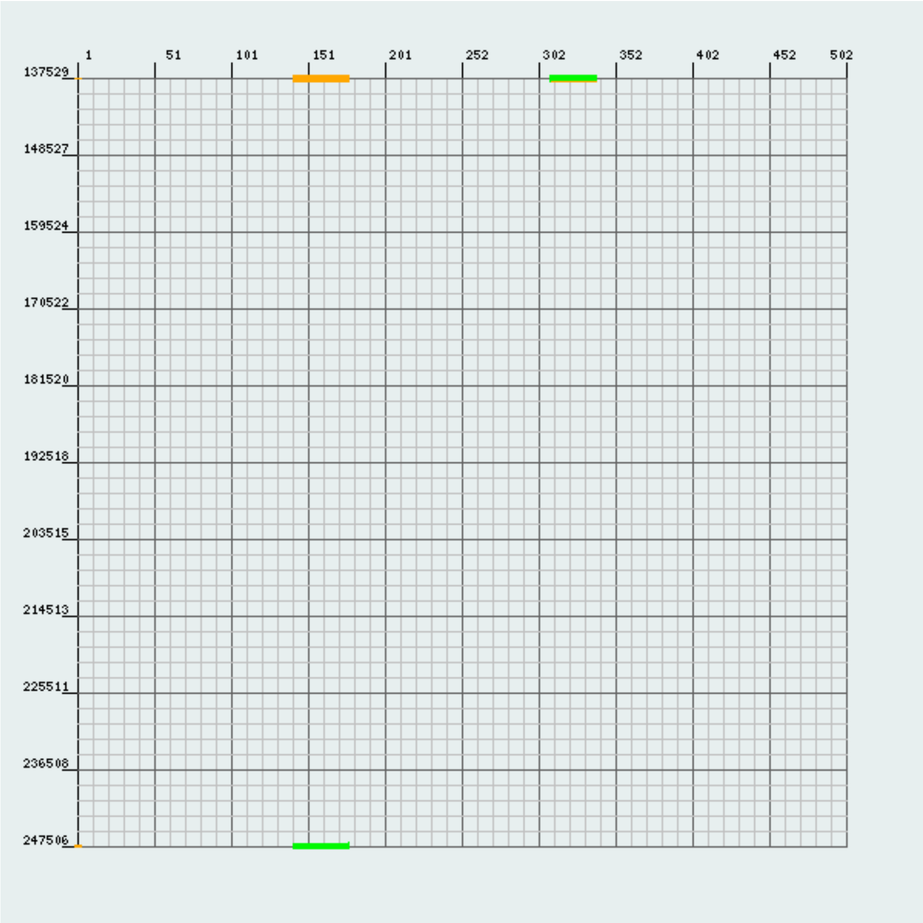

Hsp_num	0
Hsp_score	74
Hsp_bit_score	36.8409
Hsp_evalue	0.0685333
Hsp_Scaff-from	247396
Hsp_Scaff-to	247506
Hsp_hit-from	142
Hsp_hit-to	178
Hsp_Scaff-frame	-3
Hsp_hit-frame	0
Hsp_identity	12 (32.43%)
Hsp_positive	19 (51.35%)
Hsp_gaps	0

```
Scaff:   247506 WLKPGERGTAVDLPRFVAVVDKMASPAYAKAASGSWD 247396
                W +PG+R    +D+P  V ++D  A+P          WD
sbjct:      142 WNRPGDRILDIDIPMSVGIIDPRANPTQLNTVEFLWD 178
```

Hsp_align-len	37
Scaffold Seq	Seq

Hsp_num	1
Hsp_score	61
Hsp_bit_score	30.8752
Hsp_evalue	0.0685333
Hsp_Scaff-from	137529
Hsp_Scaff-to	137621
Hsp_hit-from	309
Hsp_hit-to	339
Hsp_Scaff-frame	-1
Hsp_hit-frame	0
Hsp_identity	13 (41.94%)
Hsp_positive	15 (48.39%)
Hsp_gaps	0
Hsp_align-len	31
Scaffold Seq	Seq

```
Scaff:   137621 STQPHPTPPPPTPSPLLPLPSPPPARPALRR 137529
                S     P PPPP     LLP   +P   A+   L R
sbjct:      309 SPNHQPEPPPPVTDNLLPTTTPQEAQQWLHR 339
```


© 1997-2009 The Regents of the University of California.
Genome Portal version:2.0.x code:2.0.x_4786 content:trunk_5026 gp-edge1.jgi-psf.org 03-Feb-2010 08:30:16.382 PST

U.S. DEPARTMENT OF ENERGY Office of Science

**Defline**	query
	query
**Length**	502
**Scaffold**	scaffold_136 (72906 bp) : 53074:53184 (111 bp)
**Others**	Other scaffolds hit by this sequence
**Browser**	View on browser

xstart: 1 **xend:** 502

ystart: 53074 **yend:** 53184

Rescale Reset

Color Key for BLAST Alignment Scores:

< 40 | 40-50 | 50-80 | 80-200 | >=200

Linear Graphic

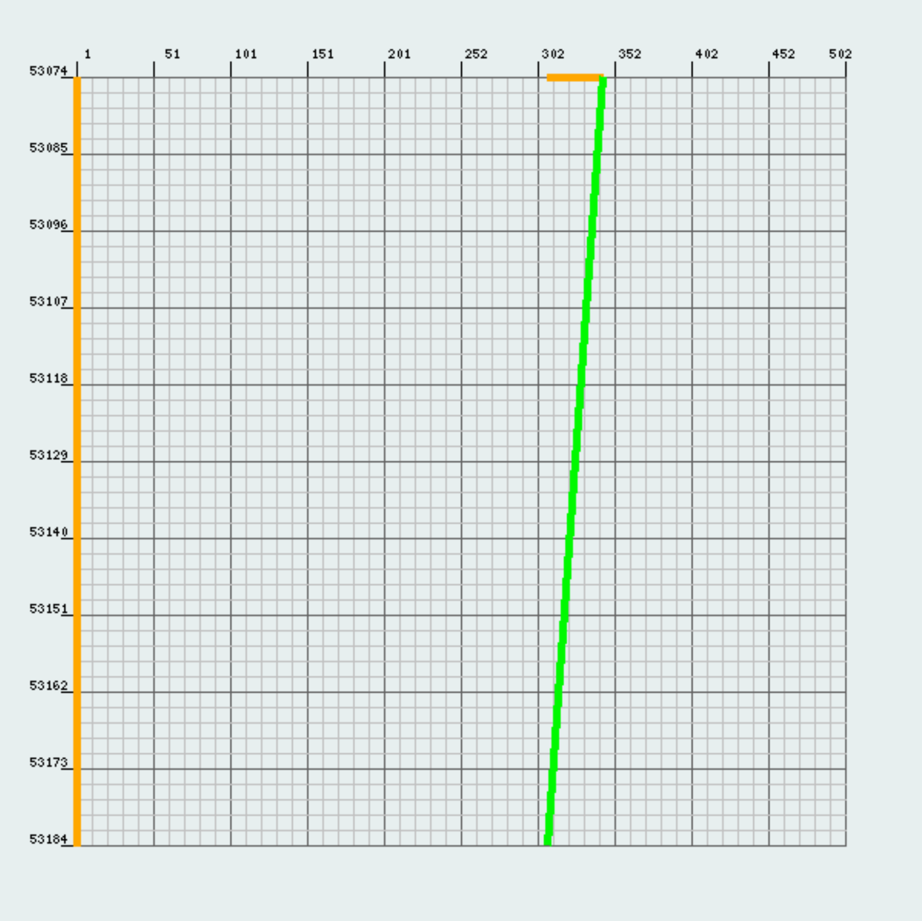

Hsp_num	0
Hsp_score	76
Hsp_bit_score	37.7587
Hsp_evalue	0.042856
Hsp_Scaff-from	53074
Hsp_Scaff-to	53184
Hsp_hit-from	308
Hsp_hit-to	344
Hsp_Scaff-frame	-1
Hsp_hit-frame	0
Hsp_identity	15 (40.54%)
Hsp_positive	20 (54.05%)
Hsp_gaps	0

```
Scaff:     53184 GYPNQQQNTPPPPTNNTSPPPTNNQQQQQQYYNPYNT 53074
                 G PN Q    PPP T+N  P   T   + QQ   + N ++T
sbjct:       308 GSPNHQPEPPPPVTDNLLPTTTPQEAQQWLHRNRFST 344
```

Hsp_align-len	37
Scaffold Seq	Seq

© 1997-2009 The Regents of the University of California.
Genome Portal version:2.0.x code:2.0.x_4786 content:trunk_5026 gp-edge1.jgi-psf.org 03-Feb-2010 08:17:09.550 PST

**Defline**	query
	query
**Length**	609
**Scaffold**	scaffold_60 (1246421 bp) : 483888:484007 (120 bp)
**Others**	Other scaffolds hit by this sequence
**Browser**	View on browser

xstart: 1 **xend:** 609

ystart: 483888 **yend:** 484007

Rescale Reset

Color Key for BLAST Alignment Scores:

< 40 | 40-50 | 50-80 | 80-200 | >=200

Linear Graphic

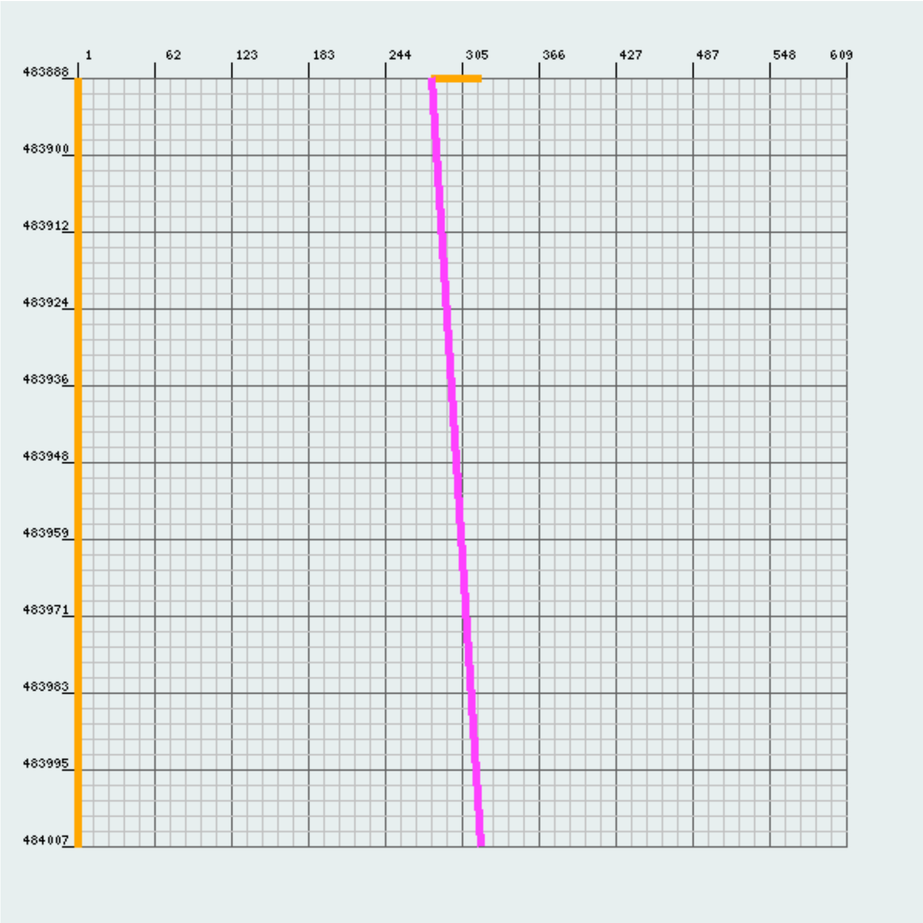

Hsp_num	0
Hsp_score	82
Hsp_bit_score	40.0314
Hsp_evalue	0.0701393
Hsp_Scaff-from	483888
Hsp_Scaff-to	484007
Hsp_hit-from	281
Hsp_hit-to	320
Hsp_Scaff-frame	3
Hsp_hit-frame	0
Hsp_identity	17 (42.50%)
[illegible]	24

```
Scaff:   483888 REIVNIVACEGLERVERAETYKQWHSRTQRAKFELLDISD 484007
                R +V +V   E    R E+ + +K WHSR    AK  +LDI+D
sbjct:      281 RSVVMVVFSEDKNRDEQLKYWKYWHSRQHTAKQRVLDIAD 320
```

Hsp_positive	24 (60.00%)
Hsp_gaps	0
Hsp_align-len	40
Scaffold Seq	Seq

© 1997-2009 The Regents of the University of California.
Genome Portal version:2.0.x code:2.0.x_4786 content:trunk_5026 gp-edge1.jgi-psf.org 03-Feb-2010 08:45:43.227 PST
