# Supplementary material for: The evolutionary diversification of LSF and Grainyhead transcription factors preceded the radiation of basal animal lineages
Source: BMC Evol Biol. 2010 Apr 18;10:101. doi: 10.1186/1471-2148-10-101 (PMC2873413; doi:10.1186/1471-2148-10-101)
Supplement: Additional file 4 — All positions in the full alignment containing gaps were deleted to produce this gap-free alignment comprising 44 characters. [file 1471-2148-10-101-S4.PDF]

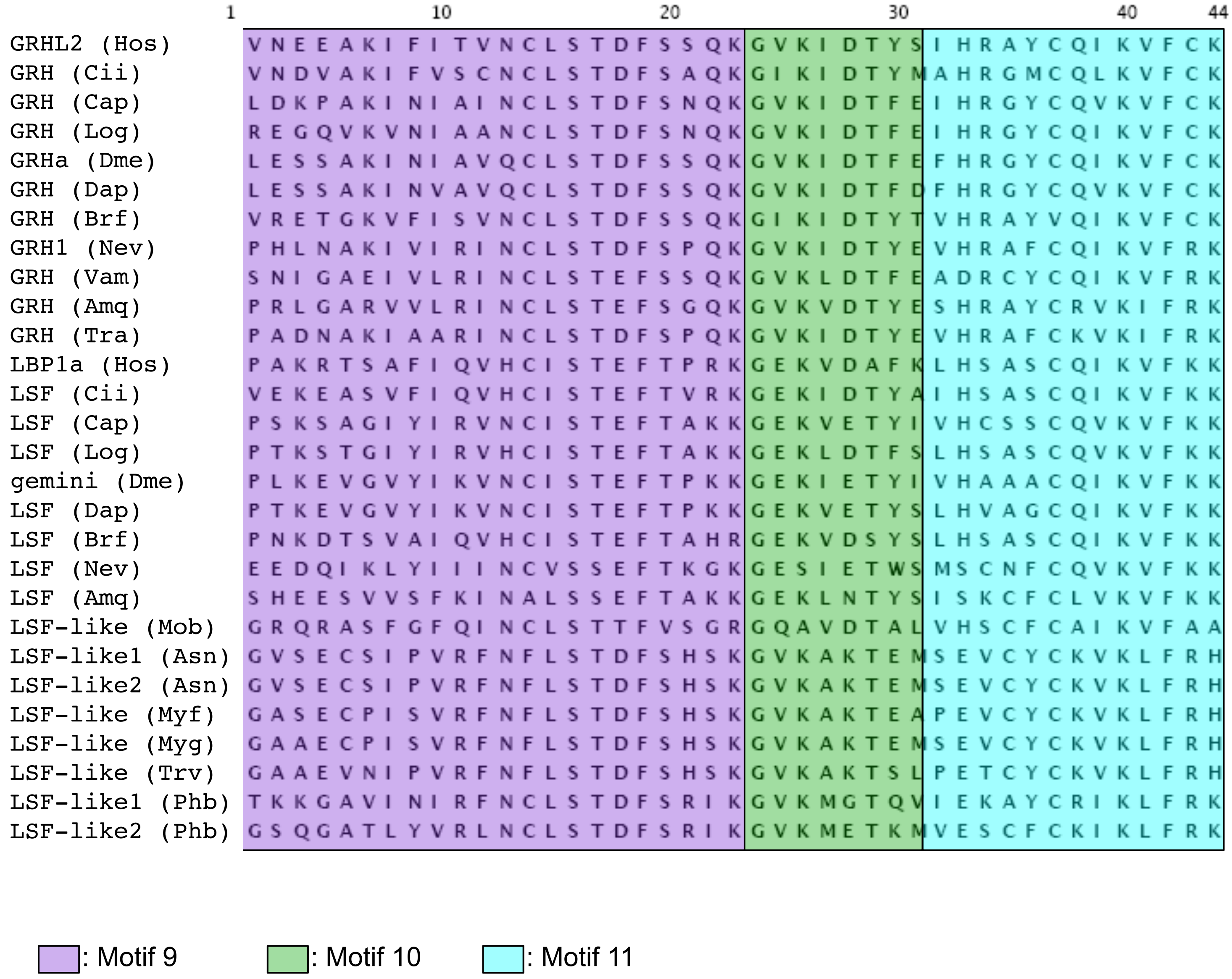

1 10 20 30 40 44
GRHL2 (Hos) VNEEAKIFITVNCLSTDFSSQK GVKIDTYS IHRAYCQIKVFCK
GRH (Cii) VNDVAKIFVSCNCLSTDFSAQK GIKIDTYM AHRGMCQLKVFCK
GRH (Cap) LDKPAKINIAINCLSTDFSNQK GVKIDTFE IHRGYCQVKVFCK
GRH (Log) REGQVKVNIAANCLSTDFSNQK GVKIDTFE IHRGYCQIKVFCK
GRHa (Dme) LESSAKINIAVQCLSTDFSSQK GVKIDTFE FHRGYCQIKVFCK
GRH (Dap) LESSAKINVAVQCLSTDFSSQK GVKIDTFD FHRGYCQVKVFCK
GRH (Brf) VRETGKVFISVNCLSTDFSSQK GIKIDTYT VHRAYVQIKVFCK
GRH1 (Nev) PHLNAKIVIRINCLSTDFSPQK GVKIDTYE VHRAFCQIKVFRK
GRH (Vam) SNIGAEIVLRINCLSTEFSSQK GVKLDTFE ADRCYCQIKVFRK
GRH (Amq) PRLGARVVLRINCLSTEFSGQK GVKVDTYE SHRAYCRVKIFRK
GRH (Tra) PADNAKIAARINCLSTDFSPQK GVKIDTYE VHRAFCKVKIFRK
LBP1a (Hos) PAKRTSAFIQVHCISTEFTPRK GEKVDAFK LHSASCQIKVFKK
LSF (Cii) VEKEASVFIQVHCISTEFTVRK GEKIDTYA IHSASCQIKVFKK
LSF (Cap) PSKSAGIYIRVNCISTEFTAKK GEKVETYI VHCSSCQVKVFKK
LSF (Log) PTKSTGIYIRVHCISTEFTAKK GEKLDTFS LHSASCQVKVFKK
gemini (Dme) PLKEVGVYIKVNCISTEFTPKK GEKIETYI VHAAACQIKVFKK
LSF (Dap) PTKEVGVYIKVNCISTEFTPKK GEKVETYS LHVAGCQIKVFKK
LSF (Brf) PNKDTSVAIQVHCISTEFTAHR GEKVDSYS LHSASCQIKVFKK
LSF (Nev) EEDQIKLYIIINCVSSEFTKGK GESIETWS MSCNFCQVKVFKK
LSF (Amq) SHEESVVSFKINALSSEFTAKK GEKLNTYS ISKCFCLVKVFKK
LSF-like (Mob) GRQRASFGFQINCLSTTFVSGR GQAVDTAL VHSCFCAIKVFAA
LSF-like1 (Asn) GVSECSIPVRFNFLSTDFSHSK GVKAKTEM SEVCYCKVKLFRH
LSF-like2 (Asn) GVSECSIPVRFNFLSTDFSHSK GVKAKTEM SEVCYCKVKLFRH
LSF-like (Myf) GASECPISVRFNFLSTDFSHSK GVKAKTEA PEVCYCKVKLFRH
LSF-like (Myg) GAAECPISVRFNFLSTDFSHSK GVKAKTEM SEVCYCKVKLFRH
LSF-like (Trv) GAAEVNIPVRFNFLSTDFSHSK GVKAKTSL PETCYCKVKLFRH
LSF-like1 (Phb) TKKGAVINIRFNCLSTDFSRIK GVKMGTQV IEKAYCRIKLFRK
LSF-like2 (Phb) GSQGATLYVRLNCLSTDFSRIK GVKMETKM VESCFCKIKLFRK
: Motif 9
: Motif 10
: Motif 11
